# Predictors of the change in bilirubin levels over twelve weeks of treatment with atazanavir

**DOI:** 10.1186/1742-6405-10-13

**Published:** 2013-05-16

**Authors:** Aoife G Cotter, Aisling Brown, Gerard Sheehan, John Lambert, Caroline A Sabin, Patrick WG Mallon

**Affiliations:** 1 HIV Molecular Research Group, School of Medicine & Medical Science, University College Dublin, Dublin, Ireland; 2 Department of Infectious Diseases, Mater Misericordiae University Hospital, Dublin, Ireland; 3 School of Medicine & Medical Science, University College Dublin, Dublin, Ireland; 4 Research Department of Infection & Population Health, UCL Medical School, London, UK; 5 Clinical Research Centre, Mater Misericordiae University Hospital, Eccles St., Dublin 7, Ireland

**Keywords:** Atazanavir, Unconjugated hyperbilirubinaemia, Bilirubin, Liver, Hepatitis C

## Introduction

Atazanavir (ATV) is a protease inhibitor used in the treatment of HIV infection. It is useful in patients on methadone replacement therapy as its once daily dosing facilitates co-administration with methadone and, unlike the non-nucleoside reverse transcriptase inhibitor, efavirenz, it does not accelerate the metabolism of methadone via induction of cytochrome P450 enzymes [1]. ATV is associated with unconjugated hyperbilirubinaemia in 6-40% of patients, overt jaundice in 7-8% and discontinuation in up to 2% [2,3]. The pathophysiology of ATV-hyperbilirubinaemia is analogous to Gilbert’s syndrome; ATV competitively inhibits UDP-glucuronyltransferase (UGT) enzymes leading to reduced glucuronidation of bilirubin and increased levels of unconjugated bilirubin [4,5]. Patients with the UGT1A1*28 genotype are particularly, but not exclusively, vulnerable [6,7]. Our study aimed to determine the clinical predictors of ATV-associated hyperbilirubinaemia in our patient population.

## Methods

We performed a single site, retrospective chart review of all patients prescribed ATV at the Mater Misericordiae University Hospital, Dublin between January 2004 and October 2007. Data collected included baseline demographic/lifestyle factors (sex, age, ethnicity, acquisition risk and methadone use), hepatitis B (HBV) and C (HCV) serology, current and previous antiretroviral therapy, baseline and week-12 routine bloods (urea and electrolytes, liver function tests , HIV-RNA, CD4+ and CD8+ T-cell counts). The last available bilirubin level for those who discontinued ATV before week-12 was utilised in the analysis.

Data was entered into a Microsoft Access database and analyses performed using SAS v9.13. Wilcoxon-Mann–Whitney tests and Spearman’s correlation were used to describe univariate associations between the factors of interest and the change in bilirubin over the first 12 weeks of ATV use. Factors considered in these analyses were: gender; age; ethnicity [Caucasian, non-Caucasian]; HIV exposure category [intravenous drug use (IVDU), non-IVDU]; smoking status [current/ex-smoker, non-smoker]; methadone use; AIDS status; CD4+ T-cell count and HIV RNA <50 copies/ml at start of ATV; antiretroviral naïve/experienced at start of ATV; nucleoside reverse transcriptase inhibitor (NRTI) included in regimen [tenofovir (TDF), non-TDF] and laboratory parameters (alkaline phosphatase (ALP), Υ-glutamyl transferase (GGT), alanine aminotransferase (ALT), alkaline phosphatase (ALP), albumin (ALB), creatinine) at the time of starting ATV, as well as the pre-ATV bilirubin level itself. Factors that were associated with a change in bilirubin in these analyses (P < 0.2) were then included in forward stepwise multivariable linear regression analyses to identify factors that were independently associated with the change in bilirubin. As the change in bilirubin was skewed, the change value was log-transformed prior to analysis; estimates were back-transformed for presentation for ease of interpretation. Due to the strong co-linearity of HCV antibody positivity, methadone use and IVDU acquisition, it was not possible to include all 3 variables in a single model; therefore the primary model included HCV antibody status and sensitivity analyses were performed after replacing this covariate in the model with IVDU acquisition or methadone use.

## Results

Ninety-three patients were prescribed ATV. Ten patients were excluded from the analysis due to self-initiated treatment interruption; these patients did not re-attend clinic after the first prescription of ATV. Four subjects discontinued ATV within 12 weeks; of these, one patient had died, one patient discontinued early due to hepatitis secondary to immune reconstitution and 2 patients stopped ATV therapy due to jaundice. 83 patients were included in the final analysis.

Baseline demographics and clinical factors are shown in Table 1. Approximately half the study group were male (55.4%), the median (IQR) age was 33 (29, 40) years, three-quarters (73.5%) were Caucasian and one-quarter were (21 (25.3%) of African ethnicity. The median (IQR) body mass index was 23 (19.5, 26.0) kg/m^2^. HIV acquisition risk was via IVDU in 61.5%, heterosexual sex in 34.9%, and homosexual sex in 3.6%. Due to the high proportion of IVDU, methadone use (44.6%) and hepatitis C virus co-infection (60.5%) were common. The majority (79 (95.2%)) were co-administered 300 mg ATV with 100 mg ritonavir, with the remainder being administered 400 mg ATV. Just under half of patients (42.2%) started TDF as part of their regimen, with 35 of the remaining 41 patients starting a regimen that contained abacavir (ABC). Median (IQR) CD4 + T-cell count, ALP, ALT and GGT were 165 (82, 264) cells/mm^3^, 79 (64, 107) IU, 35 (18, 50) IU and 52 (25, 122) IU at baseline, respectively. At baseline, 15 (18.1%) subjects had undetectable HIV RNA (<50 copies/ml) compared to 45 (54.9%) at week 12, while 46 (80.5%) had HIV RNA < 400 copies/ml at week 12.

**Table 1:** Demographic and clinical characteristics of 83 study participants, and median (IQR) change in bilirubin concentrations over the first 12 weeks of ATV treatment

	**N (%)**	**Median (IQR) change in bilirubin (μmol/L)**	***P-*value**
Gender			
Male	46 (55.4)	15 (4, 20)	0.23
Female	37 (44.6)	17 (4, 32)	
Age (years)			
Median (IQR)	33 (29, 40)		
≤ 30	27 (32.5)	16 (3, 31)	
31-40	38 (45.8)	15 (5, 21)	
> 40	18 (21.7)	17.5 (1, 23)	0.75
Ethnicity			
Caucasian	61 (73.5)	13 (4, 21)	
Non-caucasian	22 (26.5)	21 (13, 32)	0.05
Acquisition risk			
IVDU	51 (61.5)	13 (3, 20)	
Non-IVDU	32 (38.5)	21 (12, 30)	0.01
AIDS			
Yes	53 (63.9)	15 (4, 22)	
No	30 (36.1)	17 (7, 21)	0.85
Methadone use			
Yes	37 (44.6)	7 (1, 19)	0.001
No	46 (55.4)	20 (13, 26)	
Hepatitis C antibody			
Positive	46 (60.5)	10 (3, 20)	
Negative	37 (39.5)	21 (13, 31)	0.01
Hepatitis C PCR			
Positive	36 (43.4)	10 (3, 20)	
Negative	47 (56.6)	20 (7, 29)	0.02
Hepatitis B sAG			
Positive	4 (4.8)	17 (13, 27.5)	
Negative	73 (90.0)	17 (4, 22)	0.55
Treatment status at baseline			
Experienced	50 (60.2)	17 (4, 22)	0.21
Naïve	32 (38.6)	12 (2.5, 21)	
HIV RNA <50 copies/ml at baseline			
No	68 (81.9)	14 (3.5, 21.5)	
Yes	15 (18.1)	18 (13, 22)	0.31
NRTI			
TDF	42 (42.2)	11 (2, 20)	
Non-TDF	41 (50.6)	18 (19, 24)	0.02

Bilirubin levels increased from 11 (9, 14) μmol/L at baseline to 27 (17, 35) μmol/L at week-12; the median (IQR) change was 16 (4, 22) μmol/L. Five patients had elevated bilirubin at baseline (4 with grade 1 elevation, 1 with a grade 2 elevation); by week 12, all five continued to have elevated bilirubin (Table 2). In contrast, of the 78 patients with a normal bilirubin at baseline, 49 (63%) had an elevated level at week 12 (Table 2).

**Table 2:** Baseline and week-12 bilirubin grade

		**Week 12**				
**Baseline**	**N (%)**	**0**	**1**	**2**	**3**	**4**
0	78 (94.0)	27	20	21	9	1
1	4 (4.8)	2	1	1	0	0
2	1 (1.2)	0	1	0	0	0
Total	83 (100.0)	29 (35.0)	22 (26.5)	22 (26.5)	9 (10.8)	1 (1.2)

Bilirubin grade defined as per AIDS Clinical Trials Guidelines; Grade 0, normal; Grade 1, 1–1.5 × upper limit normal (ULN) (21–30 μmol/L); Grade 2, 1.5-2.5 × ULN (31–45 μmol/L); Grade 3, 2.5-5.0 × ULN (46–100 μmol/L); Grade 4, > 5 × ULN (>100 μmol/L).

Univariate analyses (Table 1) suggested that Caucasian ethnicity, TDF use, HCV antibody positivity and methadone use were significantly associated with change in bilirubin. With respect to the continuous variables that were considered, higher levels of ALT (r = −0.23, *P =* 0.04), GGT (r = −0.30, *P =* 0.005) and ALP (r = −0.43, *P* < 0.0001) at the time of starting ATV were all significantly associated with a smaller increase in bilirubin. Neither BMI nor baseline CD4 + T-cell count significantly correlated with change in bilirubin concentration (r = −0.08, *P =* 0.62; r = 0.06, *P* = 0.56 respectively). Changes in bilirubin were generally but not significantly lower in those with higher pre-ATV levels (r = −0.10, *P* = 0.37).

Of the parameters considered, the following variables (*P <* 0.05) were therefore considered for inclusion in a forward stepwise linear regression model: baseline ALP, ALT, GGT, TDF use, Caucasian ethnicity and HCV antibody status. Of these, only baseline ALP and HCV antibody positivity were independently associated with a smaller change in bilirubin (*P =* 0.04 and 0.03, respectively, Table 3) after adjusting for baseline bilirubin, higher levels of which were also associated with smaller changes after initiation of ATV. In particular, the mean increase in bilirubin was reduced by 1% per 5 IU increment in ALP, by 20% in those who were HCV seropositive, and by 2% per mmol/L increment in baseline bilirubin.

**Table 3:** Results from multivariate linear regression final models to identify factors associated with the relative change in bilirubin over the first 12 weeks of ATV treatment

	**Relative change in bilirubin**	**95% Confidence Interval**	***P*-value**
***Main model – with HCV seropositivity***			
Baseline ALP (/5 IU increase)	0.99	0.98, 1.00	0.04
TDF use at baseline	0.86	0.72, 1.00	0.10
HCV seropositivity	0.80	0.66, 0.97	0.02
Baseline bilirubin	0.98	0.96, 1.00	0.05
***Sensitivity analysis 1 – with methadone use***			
ALP at baseline (/5 IU increase)	0.99	0.98, 1.00	0.01
TDF use at baseline	0.83	0.70, 0.99	0.04
Methadone use	0.77	0.65, 0.92	0.01
Baseline bilirubin	0.98	0.96, 1.00	0.04
***Sensitivity analysis 2 – with IVDU***			
ALP at baseline (/5 IU increase)	0.99	0.98, 1.00	0.02
TDF use at baseline	0.83	0.70, 1.00	0.05
IVDU	0.85	0.70, 1.00	0.08
Baseline bilirubin	0.98	0.96, 1.00	0.02

When sensitivity analyses were performed in which HCV antibody positivity was replaced by methadone use or IVDU status, similar results were obtained. In particular, when HCV antibody positivity was replaced by methadone use, baseline ALP, TDF use and methadone use were independently associated with a smaller change in bilirubin (*P*-values of 0.01, 0.04 and 0.01, respectively), after adjusting for baseline bilirubin. When the analysis was performed with IVDU status replacing HCV antibody positivity, baseline ALP, and TDF use were independently associated with a smaller change in bilirubin concentration (*P*-values of 0.02 and 0.05 respectively). Finally, the exclusion of 2 subjects who discontinued ATV within 4 weeks due to jaundice did not alter our findings appreciably.

## Discussion

Our data demonstrate that higher baseline ALP and HCV seropositivity predict smaller changes in bilirubin after initiation of ATV. Sensitivity analyses determined that methadone use/intravenous drug use and TDF–use were independently associated with a smaller change in bilirubin.

Patients with chronic hepatitis B or C co-infection are at greater risk of drug-induced liver injury and liver enzyme elevations than those without co-infection [8,9], therefore it is plausible that they might be at greater risk of ATV-induced hyperbilirubinaemia. We have come to the opposite conclusion; those with HCV infection generally experience smaller increases in bilirubin concentrations after initiating ATV. This is the first paper that focuses on the clinical predictors of change in bilirubin level, with the majority of the literature focusing on genetic predictors. Rotger et al. assessed the utility of genotyping and hyperbilirubinaemia and found that chronic hepatitis C and hepatitis B infection were associated with higher bilirubin levels [6]. The authors suggested that pre-treatment screening for UGT1A1*28 genotype would reduce the prevalence of jaundice from 22% to 5%. However, testing is expensive and impractical in everyday practice and may result in avoidance of use of ATV in patients who would potentially benefit from its use.

On sensitivity analysis involving the substitution of intravenous drug use or methadone use for HCV seropositivity, we determined that intravenous drug use or methadone use were associated with smaller change in bilirubin at week 12. There are 2 plausible explanations: intravenous drug use history and/ or methadone use are surrogate markers for hepatitis C seropositivity in this cohort or methadone co-administration affects plasma concentrations of ATV. The latter is supported by a pharmacokinetic study of the co-administration of methadone and ATV that demonstrated significantly lower maximum concentrations (Cmax) of plasma ATV in those on concomitant methadone [10]. However, in this previous study, despite lower ATV levels, there was no difference in the therapeutic efficacy of ATV. Although we did not measure plasma ATV concentrations in this study, the previous finding of maintenance of virological efficacy is reflected in the high levels of HIV viral suppression in our cohort. Finally, we found that concomitant use of TDF is independently associated with a smaller change in bilirubin is consistent with previous data that concomitant TDF use has been shown to lower ATV levels [11].

While our study benefited from the use of longitudinal data and a representative sample of patients (drug-users versus non-drug users, Caucasian and non-Caucasian ethnicity), it was limited by its relatively small sample size and its retrospective nature. There was no data regarding compliance although the fact that majority of subjects had an undetectable viral load would suggest adequate adherence. In conclusion, we determined that HCV antibody positivity and higher baseline bilirubin were associated with a smaller increase in bilirubin after initiation of ATV. Ultimately, this data is clinically useful and may reassure prescribing physicians regarding the merit of ATV use for intravenous drug users who are co-infected with HCV, particularly in resource limited settings where regular monitoring of liver function tests may not be feasible. Further research is required to identify clinical predictors that are readily utilisable in everyday practice.

## Competing interests

ACG has received support, in the form of sponsorship to attend meetings, from Gilead Sciences Ltd., GlaxoSmithKline (Ireland Ltd), Merck, Sharp and Dohme and Bristol-Myers-Squibb Pharmaceuticals. AB, GS & JL None. CAS has received fees for consulting, membership of data safety and monitoring boards/ advisory panels, the development of educational material and speaking engagements as well as support to travel to meetings, from the following companies: Gilead Sciences Ltd., Abbott Pharmaceuticals, Janssen-Cilag, Bristol-Myers-Squibb, Merck, Sharp and Dohme and Glaxo-Smith Kline. PWGM has received support from the following; Molecular Medicine Ireland, Scientific Foundation Ireland, ViiV Healthcare, Gilead Sciences Ltd., GlaxoSmithKline (Ireland Ltd), Abbott, Merck, Sharp and Dohme and Janssen-Cilag.

## Authors’ contributions

AGC carried out data collection, statistical analysis and drafted the manuscript. AB carried out data collection, data entry and helped draft the manuscript. GS helped draft the manuscript. JL helped draft the manuscript. CAS designed the statistical methods, supervised the statistical analysis and helped draft the manuscript. PWGM conceived the study, supervised the statistical analysis and helped draft the manuscript. All authors read and approved the final manuscript.
